# Synthetic dataset of LG M50 batteries with different degradation pathways

**DOI:** 10.1016/j.dib.2024.111076

**Published:** 2024-10-28

**Authors:** Huang Zhang, Faisal Altaf, Torsten Wik

**Affiliations:** a Department of Electrical Engineering, Chalmers University of Technology, Gothenburg 41296, Sweden; b Department of Electromobility, Volvo Group Trucks Technology, Gothenburg 40508, Sweden

**Keywords:** Lithium-ion battery, Degradation mechanisms, Degradation pathways, MC 811

## Abstract

A synthetic dataset of 12 LG M50 cells was generated using physics-based models. The model parameters for this commercial NMC 811/graphite-SiOx cell were taken from multiple sources in the literature. In particular, five degradation parameters were varied from their default values as parameter sensitivity analysis. The 12 LG M50 cells were identically discharged at a 1C galvanostatic profile to 0 % state-of-charge (SoC) and then charged at a 1C constant-current and constant-voltage (CC—CV) protocol to 100 % SoC. The ambient temperature in the simulation was set to be constant at 25 °C. As a result, 4 degradation pathways were identified with 4 different configurations of interacting degradation mechanisms, i.e., solid electrolyte interphase (SEI) growth, particle cracking, lithium plating, and loss of active material. The dataset allows for the validation of battery degradation diagnosis and prognosis methods with insights into interactions between multiple degradation mechanisms. One exemplary application of validating a knee identification method can be found in Ref. [1].

Specifications Table

**Table:** 

Subject	Electrical and Electronic Engineering
Specific subject area	Lithium-ion battery degradation diagnosis
Type of data	Table. Processed data.
Data collection	Battery models: A Doyle-Fuller-Newman (DFN) model is used to simulate underlying battery states with four degradation mechanisms coupled to the DFN model in Python Battery Mathematical Modeling (PyBaMM) [2].Cycling protocol: A cycle is defined by the following steps: (1) CC discharge at 1C until 2.5 V. (2) CV discharge until current reaches the cutoff value of 50 mA. (3) Rest for 5 min. (4) CC charge at 1C until 4.2 V. (5) CV charge until the current reaches the cutoff value of 50 mA. (6) Rest for 5 min. The ambient temperature is set to be constant at 25 °C. The simulation is saved every 20 cycles in order to reduce the file size. Note that in the processed data, positive current defines discharge and negative current defines charge.
Data source location	Institution: Department of Electrical Engineering, Chalmers University of Technology. City: Gothenburg. Country: Sweden. Latitude and longitude for collected data: (57.708870, 11.974560).
Data accessibility	Repository name: Synthetic Degradation Dataset of 12 LG M50 Batteries Data identification number: 10.17632/ry6g9cc5bw.2 Direct URL to data: https://data.mendeley.com/datasets/ry6g9cc5bw/2
Related research article	H. Zhang, F. Faisal, and T. Wik, “Battery capacity knee-onset identification and early prediction using degradation curvature,” Journal of Power Sources, vol. 608, p. 234,619, 2024. https://doi.org/10.1016/j.jpowsour.2024.234619

## 1 Value of the Data

• The dataset consists of 12 synthetic LG M50 cells with their specific degradation parameters varied and then identically cycled under one constant-current and constant-voltage (CC—CV) charging protocol and galvanostatic discharging profile at 25 °C. The simulation results of these 12 cells cover 4 different degradation pathways. In particular, some cells have knees that occurred on their capacity fade curves, which significantly shortened the simulation time.
• The dataset provides insights into interactions between multiple degradation mechanisms and the evolution of degradation modes inside LG M50 cells, which can be both time-consuming and challenging to obtain from experimental data. In particular, battery internal state trajectories that lead to a knee on the capacity fade curve are useful in understanding the cause and formation process of capacity knees. Therefore, it can help optimizing battery design and manufacturing processes and further improving battery lifetime and safety.
• The dataset can be used in a range of applications, i.e., 1) validating non-invasive battery degradation diagnosis and prognosis methods with additional physical interpretation in the laboratory environment; 2) optimizing battery management systems, for example, improving onboard battery state-of-health (SoH) estimation accuracy and accelerating onboard algorithms development and validation process; 3) improving battery SoH estimation and lifetime prediction models by allowing a faster learning process without the need for complex architecture thanks to no measurement noise in the synthetic dataset.
• The dataset can also be used as a research and educational resource for battery researchers and students to gain insights into different battery degradation processes with respect to changes of their specific degradation parameters. By analyzing this dataset, large-scale synthetic datasets generation for different chemistries under a wide range of operating conditions can be promoted in the battery community.

## 2 Background

The rapid market adoption of lithium-ion batteries has significantly reduced their costs [3]. Nevertheless, understanding battery degradation processes under a wide range of usage profiles is critical for them being cost-effective in decarbonization of transportation and power sectors. The synthetic degradation data provides insights into interactions between multiple degradation mechanisms, and the evolution of different degradation modes (forming so-called degradation pathways) inside battery cells with reduced experimental burdens [4,5]. Moreover, to better validate battery degradation diagnosis and prognosis methods on a range of degradation pathways, synthetic data generated using physics-based models that complement experimental data can be very useful since the degradation mechanisms are known a priori. Here, a synthetic dataset of 12 LG M50 cells covering 4 degradation pathways has been generated using physics-based models, of which the particle cracking-induced knee pathway was used for validating the effectiveness of our proposed knee-onset and knee identification method in Ref. [1]. As a complement to the original research article [1], the full description of this synthetic dataset is provided here.

## 3 Data Description

The synthetic dataset consists of 12 LG M50 cells covering 4 degradation pathways with multiple interacting degradation mechanisms, i.e., solid electrolyte interphase (SEI) growth, particle cracking, lithium plating, and loss of active material. The technical specifications of the cells are summarized in Table 1. For each degradation pathway, 3 synthetic LG M50 cells are tested. In the following, we refer to the specific pathway (*ƿ*) by the index *x*={1,2,3,4}, and the cell (*c*) by the index *y* ={1,2,3}. The cell labels for each degradation pathway are summarized in Table 2.

**Table 1:** Technical specifications for INR21700-M50 cell [6].

Manufacturer	LG Chem
Model	INR21700-M50
Positive electrode	LiNiMnCoO2 [7]
Negative electrode	Graphite-SiOx [7]
Separator	Ceramic-coated [7]
Size (diameter × height)	21.00 mm × 70.00 mm [7]
Weight	68.38 g [7]
Nominal capacity	5 Ah
Nominal voltage	3.63 V
Charge cutoff voltage	4.2 V
Discharge cutoff voltage	2.5 V
Cutoff current	50 mA

**Table 2:** LG M50 cells in each degradation pathway.

Pathway	Cell labels
1	[p1c1,p1c2,p1c3]
2	[p2c1,p2c2,p2c3]
3	[p3c1,p3c2,p3c3]
4	[p4c1,p4c2,p4c3]

**Fig. 1:**
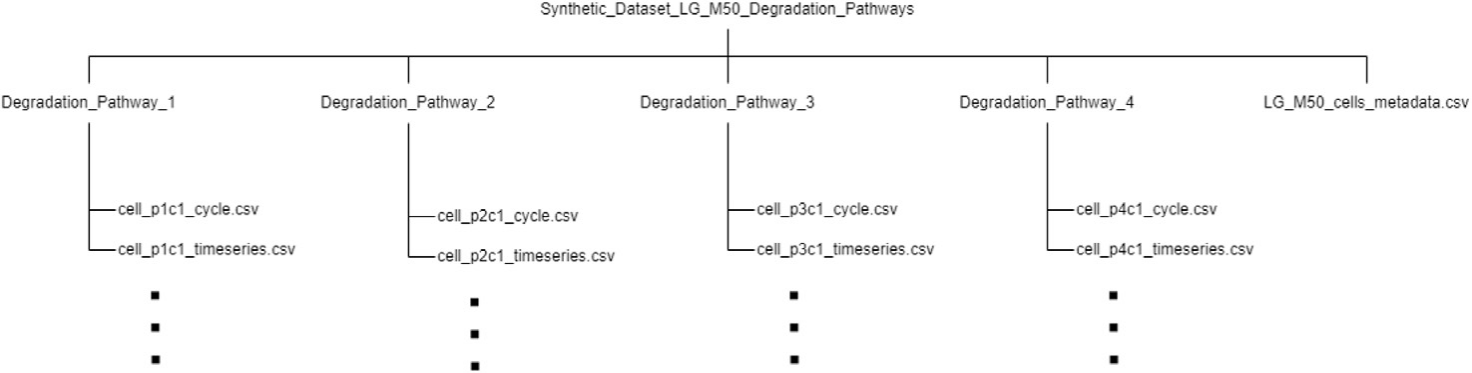
The structure of dataset files.

The overall structure of dataset files is illustrated in Fig. 1. In the parent folder named Synthetic_Dataset_LG_M50_Degradation_Pathways, one can find 1) a single file named LG_M50_cells_metadata.csv, which describes metadata for all the 12 LG M50 cells covering 4 degradation pathways, and 2) four sub-folders, one for each degradation pathway, which contain time-series and cyclic data of each cell. In each of these sub-folders, the following files can be found:

• 3 cell_p_x_c_y_cycle.csv files that store cyclic data with x and y indicating the degradation pathway and cell number, respectively.
• 3 cell_p_x_c_y_timeseries.csv files that store time-series data with x and y indicating the degradation pathway and cell number, respectively.

Note that, to reduce the size of the saved simulation file for a large number of cycles, the simulation is saved every 20 cycles. Moreover, only data related to the degradation mechanisms in this study have been extracted.

## 4 Experimental Design, Materials and Methods

To understand interactions between multiple degradation mechanisms and their resulting degradation pathways inside commercial lithium-ion cells, we generated this synthetic dataset of 12 LG M50 cells that undergo a range of degradation pathways using physics-based models. Specifically, lithium-ion battery states are simulated using a Doyle–Fuller–Newman (DFN) model [8]. Four degradation mechanisms, i.e., solid electrolyte interphase (SEI) growth [9], particle cracking [10], lithium plating [11] and loss of active material (LAM) [12], are coupled to the DFN model in Python Battery Mathematical Modeling (PyBaMM) library (version 22.9) [2].

The DFN model parameters (i.e., electrode parameters, electrolyte parameters, and separator parameters) are taken from Chen et al. [7] for a commercial NMC 811/graphite-SiOx cylindrical cell manufactured by LG Chem (INR21700 M50, 5 Ah). These LG M50 cells have a nominal capacity of 5 Ah with a lower voltage cut-off of 2.5 V and an upper voltage cut-off of 4.2 V. The degradation parameters were not measured by Chen et al. [7]. Therefore, we must turn to PyBaMM for their values. The default values provided in PyBaMM are taken from multiple sources and are listed in Table S4 and Table S5 in the supplementary information of Ref. [11]. The default values of key degradation parameters in modeling four degradation mechanisms are listed in Table 3. Furthermore, to simulate the possible knee occurrence on the capacity fade curve, three key degradation parameters corresponding to three degradation mechanisms that can cause knee occurrence are chosen to vary, i.e., cracking rate in Parisʼs law ($k_{\text{cr}}$) in modeling particle cracking, decay rate for dead lithium formation ($\gamma_{0}$) in modeling lithium plating, and loss of active anode material proportional term (β) in modeling LAM. The varying factors of these three degradation parameters are listed in Table 4 and are chosen to increase gradually so that the capacity fade can accelerate and exhibit a knee.

**Table 3:** Battery degradation parameters with their default values.

Degradation mechanism	Parameter	Default value	Unit	Ref.
SEI growth	Solvent diffusivity in Fick's law ($D_{\text{sol}}$)	$2.5\;\times \;10^{-22}$	$m^{2}\;s^{-1}$	[9]
Particle cracking	Cracking rate in Paris's law ($k_{\text{cr}}$)	$3.9\;\times \;10^{-20}$	–	[13]
Lithium plating	Decay rate for dead lithium formation ($\gamma_{0}$)	$10^{-6}$	$s^{-1}$	[11]
Loss of active material	Loss of active anode material proportional term (β)	$2.7778\;\times 10^{-7}$	$s^{-1}$	[11]

**Table 4:** Battery degradation pathways with their varied parameters.

Pathway	Coupled degradation mechanisms	Varied degradation parameter(s)	Varied factors (× default value)
1	SEI growth Particle cracking	Cracking rate in Paris's law ($k_{\text{cr}}$)	[10,30,50]
2	SEI growth Lithium plating	Decay rate for dead lithium formation ($\gamma_{0}$)	[1,5,10]
3	SEI growth Lithium plating Loss of active material	Loss of active anode material proportional term (β)	[1,10,20]
4	SEI growth Lithium plating Particle cracking Loss of active material	Cracking rate in Paris's law ($k_{\text{cr}}$) Loss of active anode material proportional term (β)	[k50β1,k30β10,k10β20]

The LG M50 cells are discharged at 1C to 2.5 V and a current cut-off of C/100 (50 mA) followed by a rest for 5 min. The cells are subsequently charged at 1C to 4.2 V and a current cut-off of C/100 (50 mA) and then allowed to rest for another 5 min. The ambient temperature is assumed to be constant at 25 °C. The step-by-step cycling protocol is described in Table 5.

**Table 5:** Description of the cycling protocol.

Step	Action	Exit condition
1	CC discharge at 1C	Voltage reaches 2.5 V
2	CV discharge	Current reaches 50 mA
3	Rest	Time reaches 5 min
4	CC charge at 1C	Voltage reaches 4.2 V
5	CV charge	Current reaches 50 mA
6	Rest	Time reaches 5 min

Finally, the simulation was set up for each synthetic LG M50 cell in PyBaMM as follows:

1. Create a DFN model coupled with specific degradation mechanisms for each pathway listed in Table 4.
2. Vary the values of three key degradation parameters for each pathway listed in Table 4.
3. Specify the type of mesh, the number of mesh points, and the type of spatial method to use on each subdomain.
4. Select the type of solver to use to solve the model.
5. Define the experimental conditions to solve the model listed in Table 5.
6. Save the simulation into pkl files at the termination of each simulation.
7. Post-process simulation and save time-series battery usage and internal state trajectories into csv files.

Note that SciPy, an open-source scientific computing library for the Python programming language [14], is used for processing the data, such as the linear correlation between knee-onset and knee in the related research article [1].

## Limitations

In total, 12 LG M50 cells were synthesized with their specific degradation parameters varied, then cycled under one constant-current and constant-voltage (CC—CV) charging protocol and one galvanostatic discharging profile. To identify more battery degradation pathways, degradation data of these LG M50 cells cycled under other charging protocols and dynamic driving profiles at different ambient temperatures are recommended to be generated in the future. Moreover, it has been found in existing studies that values of some model parameters (e.g., positive and negative solid phase diffusion coefficient and electrochemical reaction rate constant) vary significantly with the aging of the battery. Therefore, adaptive approaches to address these model uncertainties are needed for high-fidelity battery modeling and simulation.

## Ethics Statement

We confirm that we have read and followed the ethical requirements for publication in Data in Brief. The current work does not involve human subjects, animal experiments, or any data collected from social media platforms.

## CRediT Author Statement

**Huang Zhang:** Conceptualization, Methodology, Software, Validation, Formal analysis, Data curation, Writing – original draft. **Faisal Altaf:** Resources, Writing – review & editing, Supervision, Project administration, Funding acquisition. **Torsten Wik:** Resources, Writing – review & editing, Supervision, Funding acquisition.

## Data Availability

Mendeley DataSynthetic Degradation Dataset of 12 LG M50 Batteries (Original data).
